# Fractal genetics and heterogeneous phenotypes of mitochondrial disease require appropriate logistics of managing network structures

**DOI:** 10.1016/j.ymgmr.2019.100492

**Published:** 2019-07-05

**Authors:** Josef Finsterer

**Affiliations:** Neurological Department, Krankenanstalt Rudolfstiftung, Messerli Institute, Vienna, Austria

**Keywords:** Myasthenia, Acetylcholine-receptor, Autoimmunity, Antibodies, Complications

Letter to the Editor

With interest we read the article by Karaa et al. about establishing an US-based institutional network meeting the increasing needs of mitochondrial disorder(MID) patients of requiring professional diagnostic and therapeutic management [1]. We have the following comments.

A shortcoming of the study is that the term “primary mitochondrial disorders” was not defined. We should know if “primary” means disorders with defective respiratory chain functions, with defective replication/maintenance of mtDNA, with a mtDNA mutation, or disorders with mutations in any mitochondrial protein [2]. Definition of “primary” is crucial with regard to epidemiology and thus planning of the number of excellence centers within a mito-network.

A further shortcoming is that it was not defined how MIDs were diagnosed. We should know if a MID was diagnosed genetically, biochemically, upon immune-histology, and if the Walker, Bernier, or the mitochondrial disease criteria (MDC) were applied [3]. Modality of diagnosing is crucial with regard to diagnostic and therapeutic equipment of excellence centers and thus costs for establishing a network.

Since the patients' needs strongly depend on duration and severity of the disease, we should be informed about onset, duration, and severity of MIDs included. From those carrying an mtDNA variant we should know the heteroplasmy rate, since it may determine phenotype and disease trajectory [4]. Network centers need to have the possibility for follow-ups and to screen first-degree relatives.

Since MIDs are usually multisystem diseases [5], patients require multidisciplinary surveillance and management. To optimally meet the patients' needs, MIDs should be prospectively investigated for multisystem involvement even if they are initially asymptomatic. Thus, excellent centers need to provide facilities for managing multisystem disease.

Overall, MID patients require genetic diagnosing, investigations of clinically affected/unaffected first-degree relatives, prospective investigations for multisystem disease, and close follow-ups. Establishing a network/platform for patients, caregivers, and treating physicians is a first step towards optimizing MID management.

## Funding

No funding was received.

## Author contribution

JF: design, literature search, discussion, first draft, critical comments.

## Declaration of Competing Interest

None.
